# Long-Read Metagenome-Assembled Genomes Improve Identification of Novel Complete Biosynthetic Gene Clusters in a Complex Microbial Activated Sludge Ecosystem

**DOI:** 10.1128/msystems.00632-22

**Published:** 2022-11-29

**Authors:** Roberto Sánchez-Navarro, Matin Nuhamunada, Omkar S. Mohite, Kenneth Wasmund, Mads Albertsen, Lone Gram, Per H. Nielsen, Tilmann Weber, Caitlin M. Singleton

**Affiliations:** a Center for Microbial Communities, Department of Chemistry and Bioscience, Aalborg University, Aalborg, Denmark; b The Novo Nordisk Foundation Center for Biosustainability, Technical University of Denmark, Kgs. Lyngby, Denmark; c Division of Microbial Ecology, Centre for Microbiology and Environmental Systems Science, University of Vienna, Vienna, Austria; d Department of Biotechnology and Biomedicine, Technical University of Denmark, Kgs. Lyngby, Denmark; Wageningen University

**Keywords:** biosynthetic gene cluster, secondary metabolite, wastewater treatment plant, activated sludge, metagenome-assembled genome

## Abstract

Microorganisms produce a wide variety of secondary/specialized metabolites (SMs), the majority of which are yet to be discovered. These natural products play multiple roles in microbiomes and are important for microbial competition, communication, and success in the environment. SMs have been our major source of antibiotics and are used in a range of biotechnological applications. *In silico* mining for biosynthetic gene clusters (BGCs) encoding the production of SMs is commonly used to assess the genetic potential of organisms. However, as BGCs span tens to over 200 kb, identifying complete BGCs requires genome data that has minimal assembly gaps within the BGCs, a prerequisite that was previously only met by individually sequenced genomes. Here, we assess the performance of the currently available genome mining platform antiSMASH on 1,080 high-quality metagenome-assembled bacterial genomes (HQ MAGs) previously produced from wastewater treatment plants (WWTPs) using a combination of long-read (Oxford Nanopore) and short-read (Illumina) sequencing technologies. More than 4,200 different BGCs were identified, with 88% of these being complete. Sequence similarity clustering of the BGCs implies that the majority of this biosynthetic potential likely encodes novel compounds, and few BGCs are shared between genera. We identify BGCs in abundant and functionally relevant genera in WWTPs, suggesting a role of secondary metabolism in this ecosystem. We find that the assembly of HQ MAGs using long-read sequencing is vital to explore the genetic potential for SM production among the uncultured members of microbial communities.

**IMPORTANCE** Cataloguing secondary metabolite (SM) potential using genome mining of metagenomic data has become the method of choice in bioprospecting for novel compounds. However, accurate biosynthetic gene cluster (BGC) detection requires unfragmented genomic assemblies, which have been technically difficult to obtain from metagenomes until very recently with new long-read technologies. Here, we determined the biosynthetic potential of activated sludge (AS), the microbial community used in resource recovery and wastewater treatment, by mining high-quality metagenome-assembled genomes generated from long-read data. We found over 4,000 BGCs, including BGCs in abundant process-critical bacteria, with no similarity to the BGCs of characterized products. We show how long-read MAGs are required to confidently assemble complete BGCs, and we determined that the AS BGCs from different studies have very little overlap, suggesting that AS is a rich source of biosynthetic potential and new bioactive compounds.

## INTRODUCTION

The microbial world is a powerhouse of production for a range of natural products known as secondary (specialized) metabolites (SMs). SMs are small molecules that are not essential for cell growth but are important for interactions with other microorganisms and the surrounding environment (1, 2). These interactions include *in situ* intermicrobial competition, communication, and resource acquisition. Microbial SMs have important applications in biotechnology and medicine as the source of the majority of clinical antibiotics and also some pesticides and fungicides (3, 4). Antibiotics are a very important focus for SM discovery and applications (5). While antibiotic resistance is constantly rising and has become a major global concern, the development of antibiotics from SMs has slowed (6), underlining a need to prioritize the discovery of new SMs. Experimentally characterizing SMs from microbial fermentations is difficult, as many are not produced under laboratory conditions (6, 7). Determining the diversity and functions of the SMs belonging to uncultured microorganisms in microbial communities is even more problematic. Consequently, the majority of SMs are unknown, with recent estimates suggesting only ~3% of SMs detected in genome databases having characterized products (8).

SMs belong to many different structural groups, such as terpenes, ribosomally synthesized and posttranslationally modified peptides (RiPPs), nonribosomal peptides (NRPs), and polyketides (PKs) (9). Genes involved in the biosynthesis of these SMs are usually encoded in biosynthetic gene clusters (BGCs). BGCs encode the biosynthetic pathways responsible for synthesizing precursor molecules, assembling these to precursors, and finally, modifying these scaffolds with tailoring enzymes. Furthermore, they often code genes conferring resistance to the compound produced, regulation of the compound’s production, and transporters for export of the compound (10–12). Historically, the discovery of new BGCs has primarily focused on cultured bacteria and fungi, particularly in a few talented genera such as *Streptomyces* and Aspergillus (6, 13). However, mining these well-known sources results in high rediscovery rates of already known compounds (13, 14).

The uncultured majority of environmental microorganisms has huge untapped potential for the discovery of novel BGCs and, hence, novel bioactive compounds (9, 15, 16). The bioinformatic tools for BGC detection and classification, antiSMASH (17) and BiG-SCAPE (18), are used by the majority of metagenome studies and are continuously being improved. Recent applications of antiSMASH and BiG-SCAPE have revealed that there are thousands of undescribed BGCs in soil (19–22), aquatic (23–25), wastewater (26), biocrust (27), fecal (28), and host-related environments (29).

Metagenome-assembled genomes (MAGs) are usually assembled from short reads and are often fragmented, resulting in the detection of incomplete BGCs. This is especially true for BGCs coding modular enzymes (e.g., polyketide synthase (PKS) or non-ribosomal peptide synthetase (NRPS)), as their highly repetitive regions are problematic during sequence assembly (30, 31). Using long-read sequencing, it is now possible to get longer scaffolds in metagenome assemblies (27) and high-quality (HQ) MAGs (26, 28, 32). MAGs generated with long reads provide an improved blueprint for the discovery and recovery of complete BGCs (28).

We recently presented one of the most comprehensive sets of HQ MAGs retrieved from a complex microbial community, comprising over 1,000 genomes (32). The MAGs were retrieved from activated sludge (AS) wastewater treatment plants with nutrient removal and represent many of the dominant bacterial species in AS plants worldwide (33, 34). Due to limitations in automated MAG recovery from Nanopore reads, these genomes are not representative of individual strains. Instead, they represent population bins. This genome database is a source of information about key functional groups essential to nutrient cycling and recovery in the AS system. Currently, it is not known how SMs are involved in shaping the microbial ecosystem in AS plants.

Here, we provide a catalogue of SM BGCs obtained by mining 1,080 HQ MAGs representing many of the abundant and process-critical bacteria in AS plants globally. The results can be combined with other recently recovered BGCs from AS (26) and applied to further studies of AS BGCs to determine how SMs influence community structure or competition, interactions between community members and species, and potential metabolic functions. We found that using long-read-generated HQ MAGs for mining enables the retrieval of mostly complete BGCs, uninterrupted by contig borders. Furthermore, none of the retrieved BGCs showed close similarity to any characterized reference BGCs, showing that AS is an accessible source for the discovery of novel SMs.

## RESULTS AND DISCUSSION

### Nearly 4,000 complete BGCs detected in long-read-based HQ MAGs from AS.

To determine if the use of HQ MAGs generated from long-read sequencing would greatly reduce the occurrence of incomplete BGCs, we explored the BGC potential within the MiDAS genome database from 23 Danish activated sludge wastewater treatment plants with nutrient removal (32). The MiDAS genome database comprises 1,080 bacterial HQ MAGs, encompassing 578 species within 30 phyla, most belonging to uncultured and uncharacterized species. As a first metric to assess the quality of the data for BGC discovery, we investigated how many antiSMASH-detected BGCs were complete, i.e., uninterrupted by contig breaks. To be classified as a complete BGC, the core genes as well as the antiSMASH proto-clusters and their neighborhood sequence (35) (e.g., 20 kb for PKS/NRPS BGCs) were required to be entirely included within a contig sequence. Of the 4,238 total BGCs, including modular and nonmodular architecture, detected by antiSMASH, 3,714 (87.55%), were complete (see SData 1 at https://figshare.com/articles/dataset/SData1/21295287). Furthermore, 84% of the BGCs of modular architecture, i.e., NRPS, PKS, and NRPS-PKS hybrid BGCs, were complete.

In the AS MAGs, multimodular BGCs, that is NRPS, type I PKS and transAT-PKS BGCs appeared to be mostly very short, having a median of just two modules despite being uninterrupted by contig breaks (see Fig. S1 in the supplemental material). This was observed in all phyla and contrasts with the structure of the better-known multimodular BGCs, which commonly have several modules, sometimes containing up to 35 (36). However, single-module NRPS have been identified in genomic databases, with some suggested to be used for processing and transferring amino acids (37, 38). Additionally, the bacterium Photorhabdus luminescens produces indigoidine, a blue pigment, using a single-module NRPS, IndC (39). Out of the 2,523 PKS modules detected, antiSMASH classified 787 of them (31.2%) as iterative modules. These can synthesize polyketides in a noncanonical iterative manner (40). Out of the 670 BGCs with only one PKS module, only 22.2% (149) consisted of an iterative PKS module. These findings could indicate that PKs and NRPs in AS are commonly dimers, one monomer incorporated into other molecules, produce simple compounds, or are involved in amino acid processing. Additionally, research into SMs, and therefore databases such as MIBiG (41), has been focused on certain culturable taxa with large multimodular NRPS and PKS, so fewer modules may be common in uncultured bacteria.

10.1128/msystems.00632-22.1 FIG S1 Number of complete NRPS/PKS modules in multimodular BGCs, i.e., NRPS, type 1 PKS, and transAT-PKS. The bars in the box plots represent the median, the box encompasses the IQR, and outliers (beyond 1.5 times the IQR) are represented as dots. The numbers within brackets indicate the total number of multimodular BGCs detected in the phylum. Download FIG S1, SVG file, 0.01 MB. Copyright © 2022 Sánchez-Navarro et al. 2022 Sánchez-Navarro et al. https://creativecommons.org/licenses/by/4.0/ This content is distributed under the terms of the Creative Commons Attribution 4.0 International license.

### Diverse biosynthetic potential in HQ MAGs from AS.

**FIG 1:**
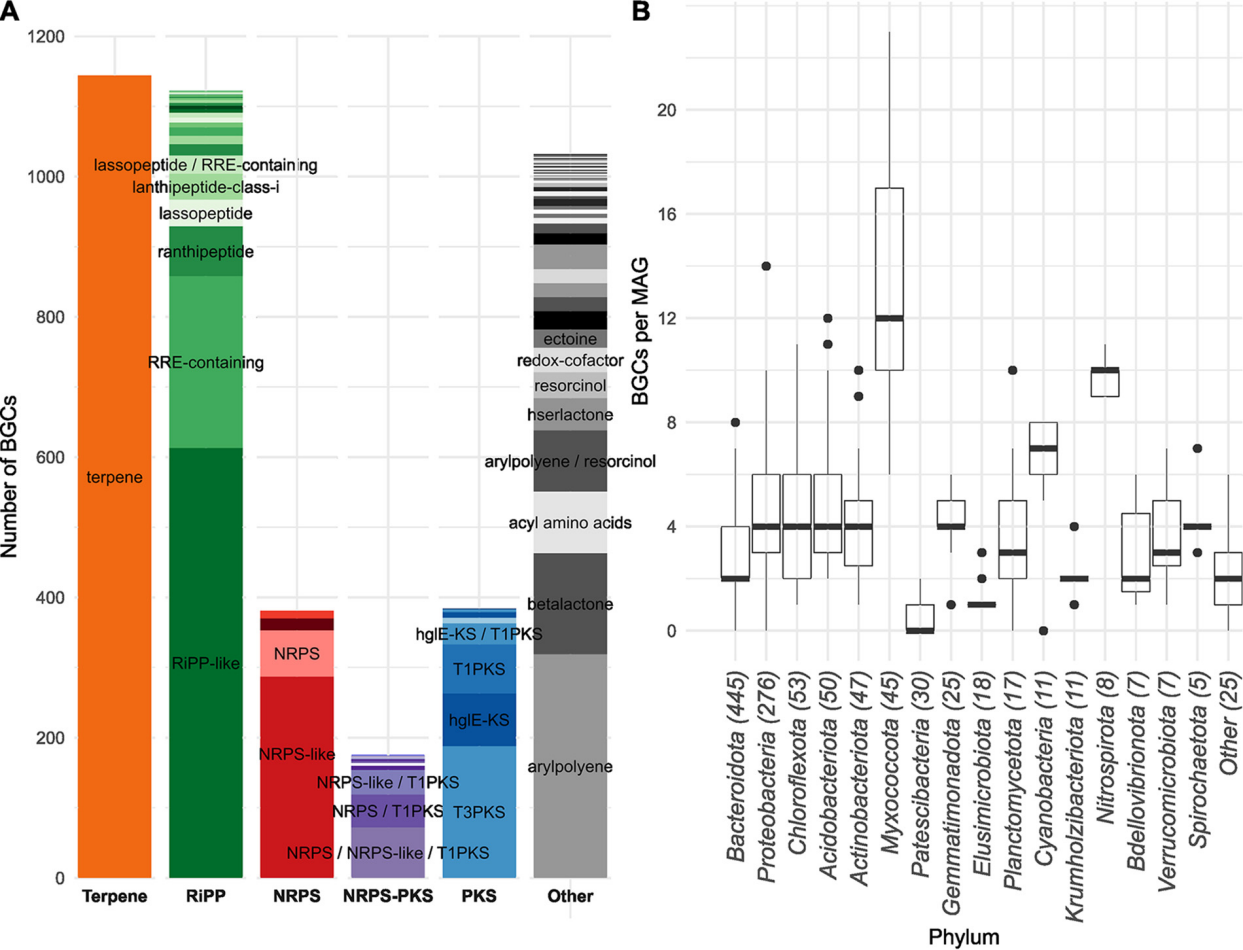
Overview of the number of BGCs detected in the HQ MAG data set. (A) Total number of BGCs found, divided into classes according to BiG-SCAPE. Data are present in SData 2 (https://figshare.com/articles/dataset/SData2/21295314) (B) Number of BGCs detected per MAG, by phylum. The numbers in the brackets represent the number of HQ MAGs. Bars represent the median, boxes encompass the interquartile range (IQR), whiskers extend to values in a range of 1.5 times the IQR, and dots are data points outside this range.

The biosynthetic potential of the 4,238 BGCs in AS was diverse, with 48 BGC types detected out of the 71 known types recognized by antiSMASH (see SData 2 at https://figshare.com/articles/dataset/SData2/21295314). The most commonly predicted products were terpenes (1,137), RiPP-like SMs (624), and aryl polyenes (399) (Fig. 1). Although PKS, NRPS, and PKS-NRPS hybrid BGCs represent most of the characterized BGCs in the MIBiG reference database, they were not commonly detected in the MAGs from AS, with only 384 PKS (9.1%) and 381 NRPS (9.0%) and 175 PKS-NRPS hybrid BGCs (4.1%) predicted.

BGCs were detected in all phyla (Fig. 1), including phyla encompassing lineages with reduced genomes, such as the *Patescibacteria* and *Dependentiae*. Most phyla had a median of four or fewer BGCs per genome. The phylum *Myxococcota* was an exception, with a median of 12 BGCs per genome. MAG GCA_016714225.1, belonging to an unknown genus in the *Myxococcaceae* family, had the most BGCs, with a total of 23, of which 20 were complete. Additionally, the *Nitrospirota* MAGs also exhibited high biosynthetic potential, with a median of 10 BGCs per genome. This phylum includes nitrite-oxidizing bacteria (NOB) and complete ammonia oxidizers (comammox), which are critical for the AS performance, but their biosynthetic potential remains mostly unexplored (see below).

**FIG 2:**
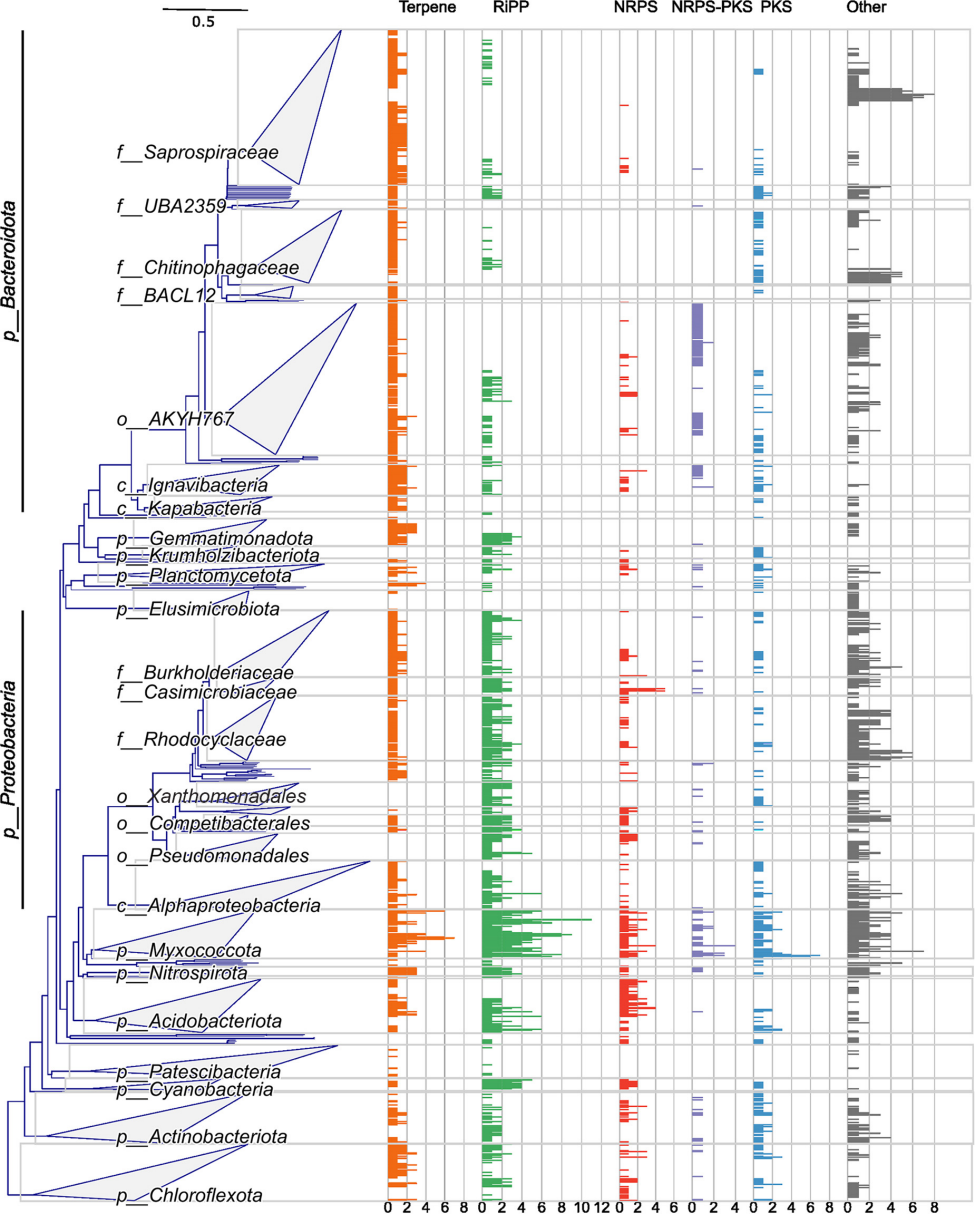
Distribution of BGCs and BGC classes across taxonomic groups. The phylogenetic tree of the 1,080 bacterial MAGs originates from GTDB-Tk and the concatenated alignment of 120 single copy proteins, trimmed to ~5,000 amino acids (aa). The bar plots represent the number of detected BGCs per MAG, by BGC class (represented also by colors). The gray boxes highlight the collapsed clades, which are also labeled. The phyla best represented by the MAGs, the *Proteobacteria* and *Bacteroidota*, have been expanded to reveal more of their diversity. The taxonomic level of the clade is indicated by the prefix; p, phylum; c, class; o, order; f, family.

Analysis of the MAGs revealed that 97.3% (1,051/1,080) contained at least one BGC. BGCs for terpene synthesis were detected in most MAGs, excluding the proteobacterial orders *Pseudomonadales* and *Xanthomonadales* (Fig. 2). Most MAGs had only one BGC for terpene synthesis; however, *Nitrospirota* contained three per MAG, and one member of *Myxococcota* contained seven (Fig. 2). Comparison of the translated terpene core BGC genes to the NCBI nonredundant (nr) database indicated that most (987/1,361) might produce uncharacterized pigments, or hopanoids, involved in cell membrane fluidity (see SData 3 at https://figshare.com/articles/dataset/SData3/21295320). RiPPs were also widespread, though notably missing in the *Flavobacteriales* and *Saprospiraceae* in the *Bacteroidota*. *Cyanobacteria* and some members of the *Myxococcota* had several RiPPs, one of them (GCA_016703425.1) containing 11. The remaining classes of BGCs were less common in the MAGs, with NRPS BGCs detected mostly in the *Acidobacteriota* and *Myxococcota*, NRPS-PKS BGCs in the *Flavobacteriales*, and PKS BGCs in the *Nitrospirota* and *Myxococcota*.

Large genome size is related to higher BGC potential in cultivated bacteria (42–44). Genome sizes of the MAGs were compared with the number of encoded BGCs, which showed a Pearson correlation of 0.62. However, this correlation was mostly driven by the phyla *Patescibacteria* and *Myxococcota*, with few and many BGCs, respectively, and removal of these groups resulted in a correlation of 0.36 (Fig. S2A). The phylum *Nitrospirota* had the most BGCs per base pair, followed by *Myxococcota* (Fig. S2B). Genome size most likely reflects the lifestyle of the MAG populations belonging to these phyla. For example, *Myxococcota* are often predatory bacteria and have complex life cycles that require large genomes (45). Isolates within the phylum are known to use a range of antibiotics and lytic enzymes as part of their epibiotic predation strategy (46). *Patescibacteria*, on the other hand, are mainly parasitic or symbiotic bacteria with streamlined genomes (47), with little need for antimicrobial agents. Though rare, a few BGCs have been detected in members of this phylum, but their role remains unknown (21).

10.1128/msystems.00632-22.2 FIG S2 Evaluation of BGC content per genome. (A) Genome size versus number of BGCs. Phyla with reduced genomes (blue) and *Myxococcota* (red) are highlighted. (B) Number of BGCs per Mbp of sequence. The bars in the box plots represent the median, the box encompasses the IQR, and outliers (beyond 1.5 times the IQR) are represented as dots. Download FIG S2, SVG file, 0.2 MB. Copyright © 2022 Sánchez-Navarro et al. 2022 Sánchez-Navarro et al. https://creativecommons.org/licenses/by/4.0/ This content is distributed under the terms of the Creative Commons Attribution 4.0 International license.

### The majority of BGCs in AS MAGs are novel.

We investigated the similarity between different BGCs detected across the HQ MAG data set. Using BiG-SCAPE, we constructed a similarity network with 6,638 edges of the 4,238 BGCs as nodes. This resulted in 2,305 connected components that were further grouped into 2,346 gene cluster families (GCFs) using a 0.3 cutoff on the edge distance (Fig. S3; see SData 4 at https://figshare.com/articles/dataset/SData4/21295329). The similarity network showed that 1,630 BGCs (~38%) were singletons, i.e., found only in one MAG, highlighting that the set of retrieved BGCs is largely nonredundant. The larger GCFs usually belonged to the more widely represented lineages in the genome set. For example, one of the largest GCFs (with 19 BGCs) consisted of BGCs of type terpene from the family *Burkholderiaceae*, which was represented by 62 MAGs. Inspection of the core genes indicated that the produced compound may be related to hopanoids, which are involved in membrane fluidity and permeability (48). The distribution of the GCFs was mostly correlated with the taxonomy assigned by GTDB (the Genome Taxonomy Database) v202. Only 141 of the GCFs were found in two or more different species, and 14 were spread across two or more genera. Similar trends were observed at higher BiG-SCAPE cutoff values (Fig. S4). BGCs belonging to the same GCF are expected to synthesize the same or closely related products. A novel BGC can be described as one that synthesizes an unknown product. Importantly, none of the BGCs were clustered into GCFs with BGCs from the MIBiG database, which is currently the gold standard repository for characterized BGCs (41). This implies that all BGCs in AS MAGs likely synthesize unknown secondary metabolites. This is a result of the BGCs in MIBiG originating predominantly from isolates, which means that the biosynthetic potential from uncultured organisms is still uncharacterized.

10.1128/msystems.00632-22.3 FIG S3 BiG-SCAPE similarity network of 4,238 HQ MAG BGCs from Singleton et al. (30) with 675 connected components and 1,630 singleton BGCs. BGC are grouped inside treemap boxes, where the area represents the number of BGCs in each BiG-SCAPE class. The colors of the nodes represent the GTDB-Tk-assigned genus based on the GTDB R202 database. Detailed information of the network and GCF can be seen in SData 10 (https://figshare.com/articles/dataset/SData10/21295416). Download FIG S3, JPG file, 0.2 MB. Copyright © 2022 Sánchez-Navarro et al. 2022 Sánchez-Navarro et al. https://creativecommons.org/licenses/by/4.0/ This content is distributed under the terms of the Creative Commons Attribution 4.0 International license.

10.1128/msystems.00632-22.4 FIG S4 Number of GCFs present in MAGs from different taxa at different levels and cutoff values. Taxonomy was assigned by GTDB207. Download FIG S4, SVG file, 0.01 MB. Copyright © 2022 Sánchez-Navarro et al. 2022 Sánchez-Navarro et al. https://creativecommons.org/licenses/by/4.0/ This content is distributed under the terms of the Creative Commons Attribution 4.0 International license.

Further, we queried the presence of similar BGCs in the genomes from public databases. The recently released BiG-FAM database contains 1.2 million BGCs from publicly available genomes and metagenomes grouped into 29,955 different GCFs (49). We compared the 4,238 BGCs detected in the HQ MAG database against the BiG-FAM database using BiG-SLiCE (50). A total of 1,477 (~35%) of the BGCs were assigned to 139 GCF models from the BiG-FAM database (BiG-SLiCE run 6, using a *T*-value cutoff of 900 for membership) (Fig. S5; see SData 5 at https://figshare.com/articles/dataset/SData5/21295338). The majority of the hits belong to terpene (*n* = 547) and RiPP-like (*n* = 535) products. Furthermore, 594 (~14%) are assigned to 8 GCFs with relatively large member size (>10,000), suggesting they might share common features that are indistinguishable by the algorithm. Out of the remaining 761 BGCs (~21%), only 353 (~8%) can be assigned to 36 GCF models with MIBiG entries as their core member. Overall, the similarity network analysis showed that the BGCs identified in the AS microbiota were largely unique and distinct from other BGCs in BiG-FAM, which include 20,584 MAGs (49).

10.1128/msystems.00632-22.5 FIG S5 BiG-SLICE query assigned 1,477 BGCs to 139 GCF models from the BiG-FAM database. Square nodes represent GCF models and their ID, while circular nodes represent BGCs from Singleton et al. (30), color-coded by BiG-SCAPE class. (A) Out of 1,477 BGCs, 924 can be assigned to 41 GCF models with known MIBiG BGCs as core members. (B) Meanwhile, 553 BGCs can be assigned to 98 GCF models with no known BGCs as core members. Detailed information on each BGC query and GCF models can be seen in SData 11 (https://figshare.com/articles/dataset/SData11/21295419). Download FIG S5, SVG file, 1.8 MB. Copyright © 2022 Sánchez-Navarro et al. 2022 Sánchez-Navarro et al. https://creativecommons.org/licenses/by/4.0/ This content is distributed under the terms of the Creative Commons Attribution 4.0 International license.

### BGCs in process-critical bacteria.

**FIG 3:**
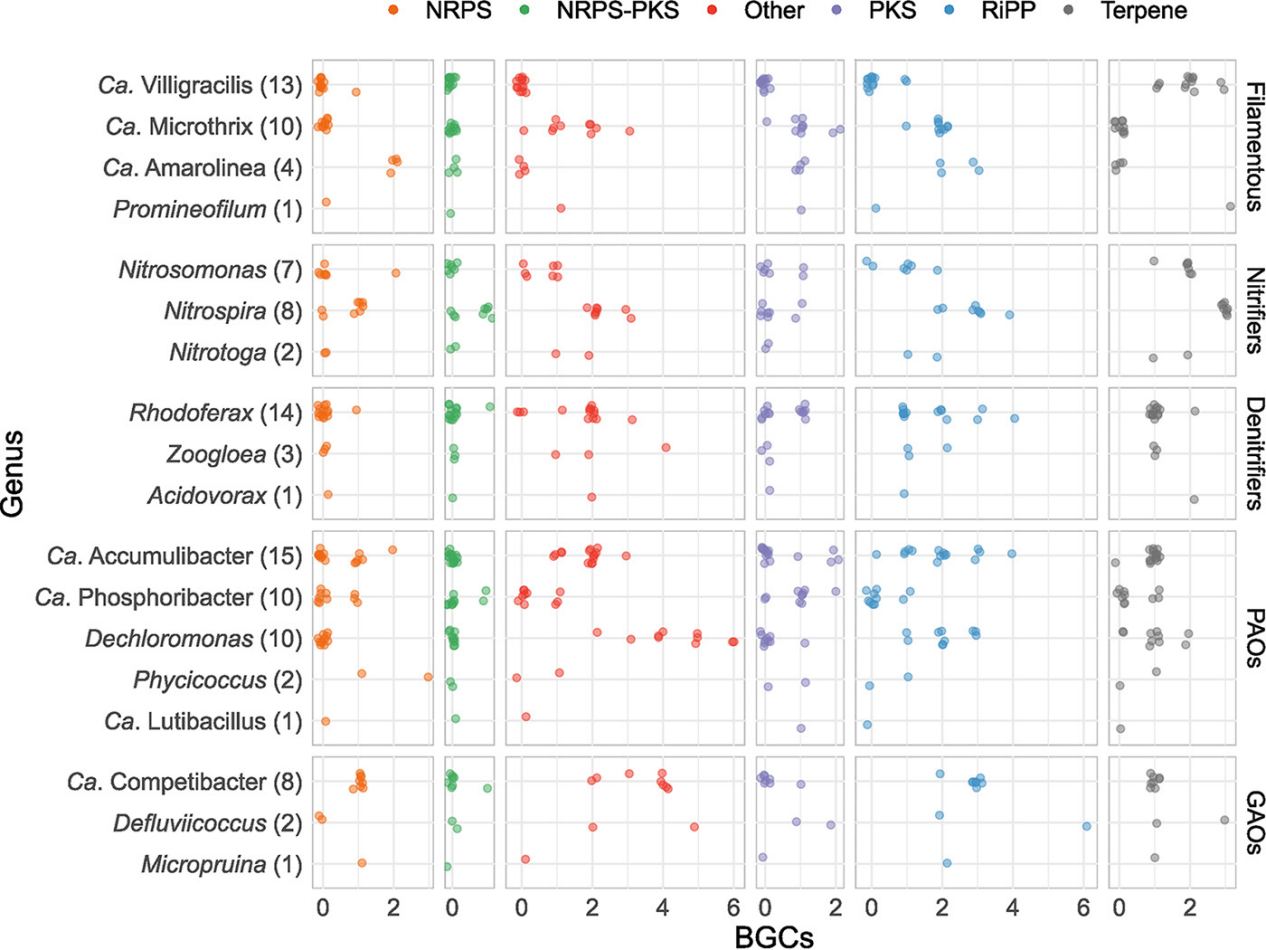
BGCs in selected genera functionally relevant and/or abundant in WWTPs with nutrient removal. Taxonomy is indicated on the *y* axis, with the number of BGCs indicated on the *x* axis. Functional group is indicated on the right. The number of MAGs in the group examined is indicated in the parentheses.

Several genera belonging to abundant and process-critical bacteria in AS showed large and varied biosynthetic potential (Fig. 3). Filamentous bacteria can cause foaming and severely undermine wastewater treatment efficiency (51). Among the common and often abundant filamentous bacteria, two genera, “*Candidatus* Villigracilis” and “*Ca.* Promineofilum,” possess terpenes, but almost no RiPPs. “*Ca.* Microthrix” and “*Ca.* Amarolinea” have several RiPPs but no terpenes, which is unusual in our data set, as most MAGs encoded BGCs for terpenes. NRPS BGCs were only detected typically in “*Ca*. Amarolinea,” while “*Ca.* Promineofilum,” “*Ca.* Microthrix,” and “*Ca*. Amarolinea” encoded PKS BGCs.

Among the nitrifiers, RiPPs and terpenoid BGCs could be detected in all genera. BGCs for homoserine lactone synthesis, common quorum sensing autoinducers (52), were detected in all three nitrifier genera. NRPS and NRPS-PKS hybrid BGCs hybrids were detected only in *Nitrospira*, but not in *Ca*. Nitrotoga or the ammonia-oxidizing bacteria (AOB) *Nitrosomonas*. All denitrifiers investigated showed a fairly similar biosynthetic potential, generally with one or two BGCs for terpenes, one RiPP, and no NRPS or PKS BGCs.

Among polyphosphate-accumulating organisms (PAOs), the most abundant genera in wastewater treatment plants (WWTPs), “*Ca.* Phosphoribacter” and “*Ca*. Lutibacillus” (53), showed little biosynthetic potential, in contrast to *Dechloromonas* (mean of 7.6 BGCs, up to 10) and “*Ca.* Accumulibacter” (mean of 5.6 BGCs, up to 10), which were rich in BGCs. These two genera also showed some variability in their biosynthetic potential within the genera. Within the glycogen-accumulating organisms (GAOs), *Defluviicoccus* and “*Ca.* Competibacter” showed the highest biosynthetic potential. One *Defluviicoccus* MAG (GCA_016712595.1) had a total of 14 BGCs (Fig. 3). Overall, SMs appear to be relevant to the life strategies of many process-critical bacteria, possibly for biofilm or floc formation, quorum sensing, and intermicrobial competition, but ultimately their functions are completely unknown.

### BGCs in *Nitrospira* and *Myxococcota*.

We selected two phyla of particular interest due to their high BGC potential and important roles in the AS environment, the *Myxococcota* and *Nitrospirota*, and compared their mined BGCs to those from genomes in the Genome Taxonomy Database (GTDB-R202) (Fig. S6). The GTDB MAGs originate either from GenBank or the NCBI curated RefSeq database, and GTDB classifies the MAGs with HQ or MQ (medium quality) labels following the MiMAG standards for genome completeness and contamination (54). The *Nitrospirota* AS MAGs clearly had fewer BGCs on a contig edge (2.8%) than both the HQ (39.2%) and MQ (72.8%) genomes in GTDB and were comparable with RefSeq genomes classified as complete (0%) (Fig. S6; see SData 6 at https://figshare.com/articles/dataset/SData6/21295350). *Myxococcota* AS MAGs also had fewer BGCs on a contig edge (13.6%) than both the HQ (57.6%) and MQ (71.6%) genomes in GTDB (Fig. S6; see SData 7 at https://figshare.com/articles/dataset/SData7/21295392). As long-read sequencing has only recently facilitated large MAG recovery efforts, the majority of MAGs in GenBank are generated from short-read data. We chose to use HQ MAGs and genomes originating from RefSeq for BGC comparisons within these phyla.

10.1128/msystems.00632-22.6 FIG S6 Quality assessment of detected BCGs for the phyla *Myxococcota* (left) and *Nitrospirota* (right). AS HQ MAGs comprise MAGs in this study, the HQ and MQ columns are based on MIMAG (Minimum Information about a MAG) completeness and contamination standards, retrieved from GTDB. “Complete” indicates that the assembly level is “complete” or “chromosome” according to NCBI. Sample sizes are labelled under the box plots. The bars in the box plots represent the median, the box encompasses the IQR, and outliers (beyond 1.5 times the IQR) are represented as dots. Download FIG S6, SVG file, 0.03 MB. Copyright © 2022 Sánchez-Navarro et al. 2022 Sánchez-Navarro et al. https://creativecommons.org/licenses/by/4.0/ This content is distributed under the terms of the Creative Commons Attribution 4.0 International license.

**FIG 4:**
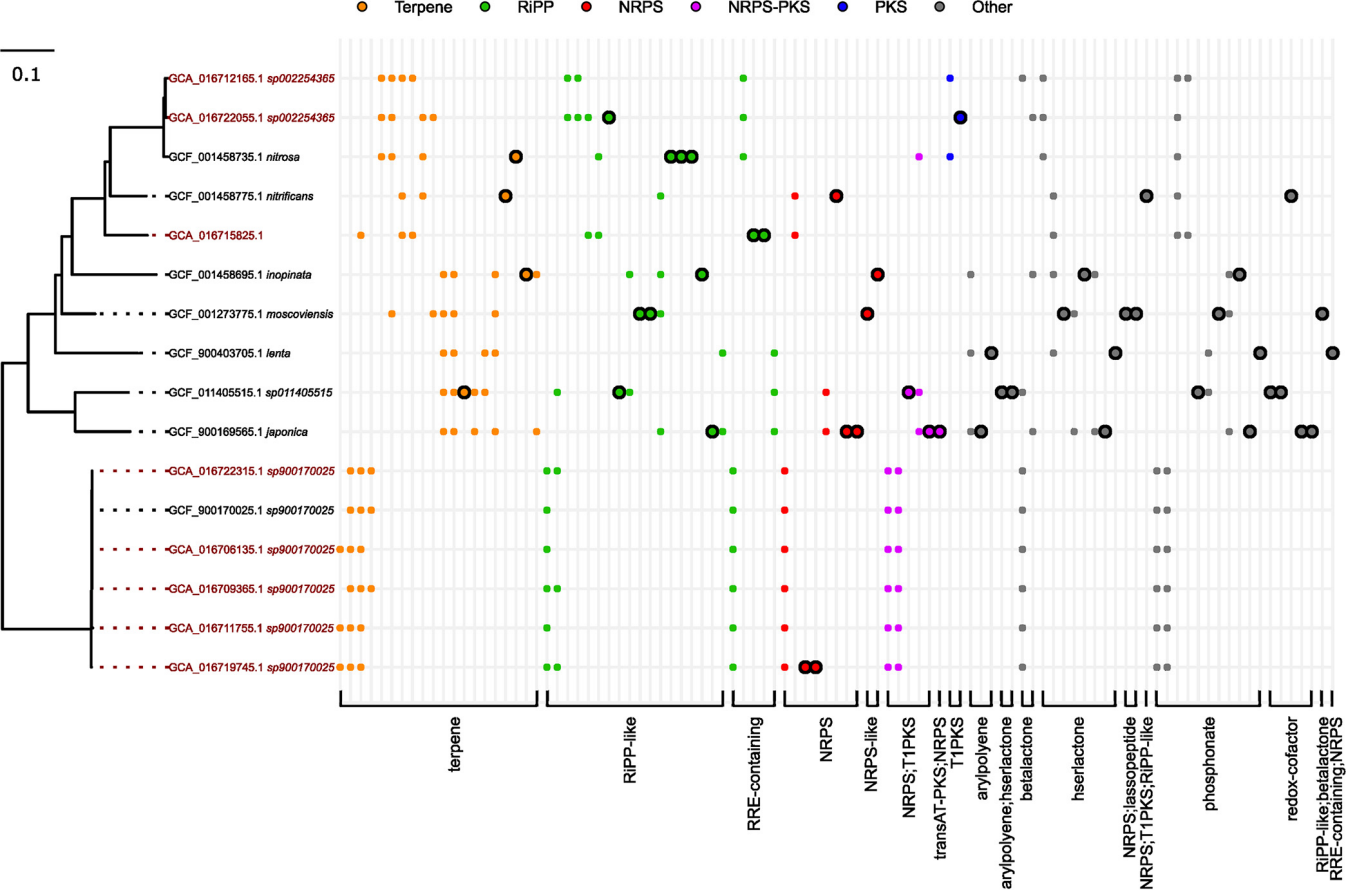
Distribution of the presence of GCFs across the phylogenomic tree of 16 genomes of *Nitrospira*. A phylogenomic tree of *Nitrospiraceae*, including eight MAGs from this study (red) and eight publicly available HQ genomes (black) was constructed using GTDB-Tk and the concatenated alignment of 120 single copy proteins trimmed to ~5,000 aa. Accession numbers are provided. Prefixes “GCA_” and “GCF_” correspond to the GenBank and RefSeq assemblies, respectively. Each column of the presence/absence matrix corresponds to a GCF (colored according to BGC class) detected using a cutoff of 0.3 on the raw_distance metric of BiG-SCAPE. Circles outlined in black indicate singletons, i.e., BGCs that are unique.

The genus *Nitrospira* incorporates the most abundant NOB in AS and is central to the nitrifying function of WWTPs. In AS, *Nitrospira* includes the canonical NOB, N. defluvii and the two comammox species, “*Ca*. Nitrospira nitrosa” and N. inopinata, none of which have been investigated for their biosynthetic potential. To assess the novelty and diversity of the BGCs detected in the family *Nitrospiraceae*, we compared GCFs in our MAGs with the genomes available in NCBI RefSeq (via GTDB-R202) for this family (Fig. 4). The majority (96.0%) of GCFs detected in our MAGs were complete, as also observed in the reference genomes (97.2%) (Fig. S6).

The biosynthetic potential of all *Nitrospira* (GTDB taxonomy) was strikingly similar (Fig. 4; SData 8) (https://figshare.com/articles/dataset/SData8/21295398). Several BGCs for terpene synthesis were detected in each genome, and all genomes contained several clusters for RiPPs. “*Ca*. Nitrospira nitrosa“ was the only species with PK clusters. Aryl polyenes, SMs that commonly function as pigments (55), were found only in the species *N. inopinata*, Nitrospira lenta, N. sp011405515, and Nitrospira japonica. Homoserine lactones are molecules that commonly function as quorum sensing autoinducers and play a central role in AS communities, as their concentration is correlated with efficient N removal (56). Interestingly, homoserine lactones were detected in all genomes from comammox bacteria (*N. inopinata* and “*Ca*. N. nitrosa”) and in N. sp011405515, supporting the previously observed production of homoserine lactones in *Nitrospira* (57). All *Nitrospira* MAGs also encoded phosphonate clusters, similar to the *Nitrospirota* MAGs recovered from soil (21). Phosphonates may provide a method for phosphorus storage (58), which is pertinent to the AS system and may suggest that *Nitrospira* is more involved in phosphorus cycling than previously thought. Overall, our BGC recovery from the AS community using HQ MAGs appeared accurate based on the similarity of BGCs with the available reference genomes.

The highest biosynthetic potential in our data set was found in the MAGs from *Myxococcota*, and in total, 86.4% of their BGCs were complete (Fig. S6; see SData 7 at https://figshare.com/articles/dataset/SData7/21295392). The biosynthetic potential of *Myxococcota* has been investigated in several isolates. Several cultured isolates have SMs that have been characterized (59), which include a variety of known antibiotics (60). BGCs were similarly enriched in *Myxococcota* from an AS sample from the Shatin WWTP (Hong Kong, China), supporting suggestions that uncultured members of the phylum are a good focus for natural product discovery (26, 59, 61). Furthermore, targeting novel genera within the *Myxococcales* is predicted to result in greater discovery than investigating different strains or species of already characterized genera (62), as intragenus SM diversity in general has been found to be relatively conserved (Fig. S4) (8).

**FIG 5:**
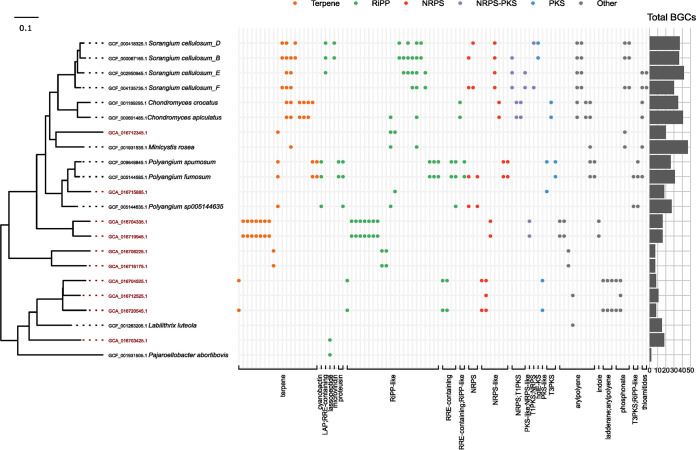
Distribution of the presence of GCFs across the phylogenomic tree of 22 genomes within the *Polyangiaceae*. Ten HQ MAGs from this study (red) and the 12 RefSeq genomes available for this family (black) are included. The phylogenomic tree on the left is a subsection from GTDB-Tk. Accession numbers are provided. Prefixes “GCA_” and “GCF_” correspond to the GenBank and RefSeq assemblies, respectively. The gray boxes indicate genus boundaries. Singletons have been removed from the matrix. The bar plot on the right represents the total number of BGCs detected.

Among the families within the *Myxococcota* phylum represented by our MAGs, *Polyangiaceae* (order *Polyangiales* in GTDB, *Myxococcales* in NCBI) was the best represented by RefSeq genomes (Fig. S7), which are excellent targets for mining BGCs. Therefore, we selected this family to compare their BGCs with those of reference genomes (Fig. 5). The *Polyangiaceae* include Minicytis rosea, a bacterium possessing the largest genome found to date, of 16 Mbp, which contains 47 BGCs (63). Only 1 of the 10 AS MAGs in this family could be assigned to a GTDB genus, underlining the novelty within this family in the AS system. Some of the GCFs in this data set were widespread and detected in distant genera. For example, the RiPP GCF 4797 was detected in *Sorangium*, *Minicystis*, and *Polyangium*, and the NRPS GCF 1266 was detected across *Sorangium* and *Polyangium* (Fig. 5; see SData 9 at https://figshare.com/articles/dataset/SData9/21295404). However, generally, our MAGs shared few or no BGCs with the selected reference genomes. MAGs GCA_016712345.1 and GCA_016715885.1 encode mostly singletons (20 singletons out of 24 BGCs, and 17 out of 24, respectively), i.e., GCFs comprising a single BGC, despite having closely related reference genomes that share GCFs among themselves (Fig. 5). This demonstrates the great potential in mining HQ MAGs from complex microbial communities for the discovery of novel GCFs compared to GCFs from cultured representatives. Interestingly, our MAGs had lower BGC counts than the most closely related genomes available in RefSeq. This was especially evident for the AS *Polyangium* MAG, which had fewer BGCs than the *Polyangium* genomes of populations isolated from soils (Fig. 5). This could be a product of the AS environment, as fewer BGCs have also been detected in *Myxococcota* from anoxic freshwater than from soil environments (61).

10.1128/msystems.00632-22.7 FIG S7 Total number of BGCs in genomes available in GTDB from families within the phylum *Myxococcota* represented in our MAG dataset. The bars in the box plots represent the median, the box encompasses the IQR, and outliers (beyond 1.5 times the IQR) are represented as dots. Download FIG S7, SVG file, 0.04 MB. Copyright © 2022 Sánchez-Navarro et al. 2022 Sánchez-Navarro et al. https://creativecommons.org/licenses/by/4.0/ This content is distributed under the terms of the Creative Commons Attribution 4.0 International license.

### Comparative study highlights the need for HQ MAGs from long-reads.

**FIG 6:**
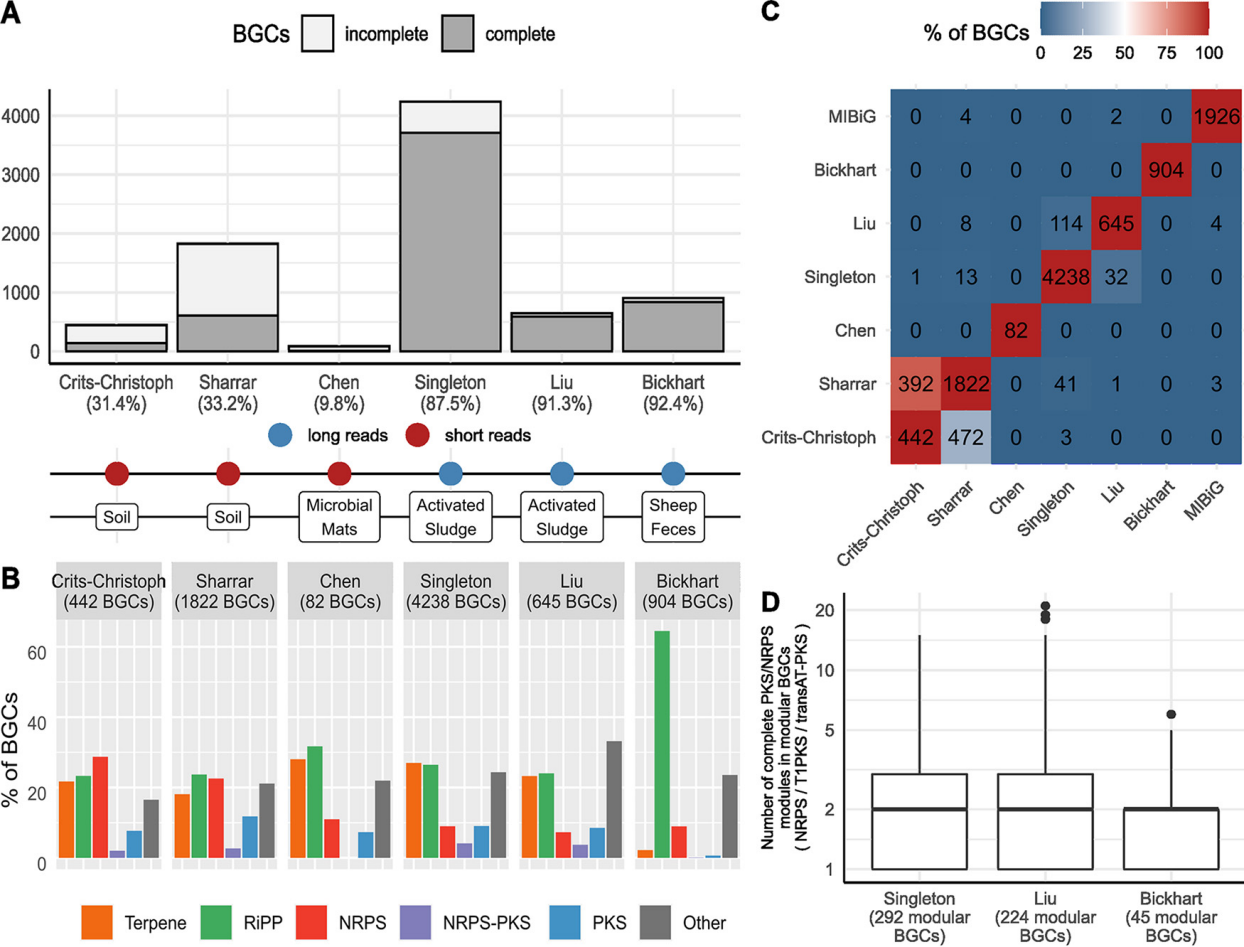
Comparison of BGC mining studies in HQ MAGs. (A) Number of complete and incomplete (on a contig border) BGCs (SData 8) (https://figshare.com/articles/dataset/SData8/21295398). (B) Proportion of detected BGCs, by BGC class. (C) Number of BGCs of one study (*x* axis) found in a GCF of another study (*y* axis), at a BiG-SCAPE cutoff value of 0.3. Color indicates the percentage of the total number of BGCs in a study. (D) Number of complete PKS/NRPS modules detected in BGCs with multimodular architecture. The bar indicates the median, the boxes indicate the interquartile range (IQR), whiskers represent a range of 1.5 times the IQR, and dots are data points outside this range. Studies examined: Crits-Christoph et al. (22), Sharrar et al. (21), Chen et al. (24), Singleton et al. (32), Liu et al. (26), Bickhart et al. (28).

To assess the effect of different sequencing technologies and ecosystems on the recovery of BGCs in environmental samples, we investigated five studies that mined for BGCs in MAGs and compared their results to our study. These studies include short-read data metagenomes from soil (21, 22) and microbial mats (24) and long-read data metagenomes from activated sludge (26) and sheep feces (28). To ensure comparability between these data sets and our own, we compared only the bacterial MAGs that met the high-quality completeness and contamination cutoffs, and we applied the same workflow parameters (see SData 10 at https://figshare.com/articles/dataset/SData10/21295416). The prevalence of biosynthetic potential observed in AS in this study (97.3% of our MAGs contain BGCs) agrees with the results of Liu et al. (26) (96.1%). This ubiquity of BGCs seems to be a common occurrence in microbial communities, as all studies detected at least one BGC in most MAGs (81.6%, 94.5%, 90.5%, and 86.3% in Sharrar et al. [21], Crits-Christoph [22], Chen et al. [24], and Bickhart et al. [28], respectively). The comparison revealed that mining for BGCs using MAGs assembled from short-read data results in mostly incomplete BGCs (>66%) (Fig. 6). However, recovering HQ MAGs from the extremely complex microbial communities of soil is particularly challenging (64), no matter the sequencing technology used (19). In HQ MAGs obtained from long reads, mostly uninterrupted BGCs were detected. The small differences in the proportion of complete BGCs could be due to the different long-read technologies used (Nanopore in Singleton et al. [32] and Liu et al. [26] and PacBio in Bickhart et al. [28]), different sequencing depths and coverage cutoffs, or differences in the proportion of modular BGC classes within genomes.

Clear differences in the proportion of BGCs recovered could be observed in relation to the ecosystem (Fig. 6). NRP BGCs were more frequently detected in studies of soil. In the study of sheep feces, most of the BGCs recovered were RiPPs, which along with the low number of modular PKS BGCs detected in this study, could affect the proportion of complete BGCs.

A BiG-SCAPE clustering of the six studies and the MiBIG database (Fig. 6) at a cutoff value of 0.3 revealed that the vast majority of BGCs recovered in all studies are novel. Only the studies by Sharrar et al. (21) and Liu et al. (26) had BGCs belonging to a previously characterized GCF. Studies coming from related environments shared some of their biosynthetic potential. A wide overlap was observed between the two soil studies (Crits-Christoph et al. [22] and Sharrar et al. [21]). These studies shared 248 GCFs, representing 88.7% and 25.9% of their BGCs, respectively. Our study shared 30 GCFs with the other study in AS, Liu et al. (26). However, these GCFs contained only 2.7% of our BGCs. The low degree of overlap between these two studies implies that the recovery of the complete biosynthetic potential in AS would require further extensive sampling and long-read sequencing of WWTPs. The rest of the studies did not show a significant overlap. At cutoff values of 0.4 and 0.5, similar results were observed (Fig. S8).

Across all studies, the detected multimodular BGCs (NRPS, type I PKS and trans-AT PKS) were very short (see SData 11 at https://figshare.com/articles/dataset/SData11/21295419). As short-read studies cannot consistently capture these multimodular repetitive regions, we did not include them in this analysis. In the long-read studies, multimodular BGCs had a median of only two modules (Fig. 6), far shorter than most of the characterized BGCs of these types. This suggests that in these ecosystems (AS, sheep feces), modular BGCs are mostly short.

**Conclusion and future perspectives**. Uncultured environmental microorganisms have huge potential for SM discovery and yet are understudied, despite the urgency of increasing resistance to current antibiotics and pesticides and the associated risks to humans and agriculture (9, 16). AS is an important resource worldwide, mostly for cleaning water and protecting human and environmental health, but it is also increasingly valued for nutrient and water recovery and its contribution to the desired circular economy. The complex, predominantly uncultured, microbial community responsible for the AS process has a plethora of novel BGCs and represents an accessible source for future characterization. While SMs have immediate importance to human health, it is likely they also have an integral role in environmental health and the function of effective wastewater treatment. This role is indicated by the prevalence of BGCs in microbial functional guilds, such as the nitrifiers, and biosynthetically talented yet uncharacterized populations within the *Myxococcota*. HQ MAGs generated from long-read data greatly improve the recovery of complete BGCs, facilitating genome mining and providing a gold standard genomic foundation for further studies. However, since these genomes do not represent individual clonal strains but, instead, composite population bins, manual curation of the BGC sequences is needed prior to experimental work. Laborious attempts to culture these populations should be preceded by *in situ* screening, as many genes may be silent under laboratory or growth conditions (7). Exciting developments in the extraction and expression of BGCs from metagenomes suggests a potential high-throughput approach for product characterization (25, 65), though product detection remains challenging. Applying metatranscriptomics to narrow down potential targets highly expressed *in situ* could increase the chances of success.

## MATERIALS AND METHODS

### Data set collection.

We selected 1,080 bacterial HQ MAGs from the 1,083 HQ MAGs from AS presented in our earlier study (see SData 1 at https://figshare.com/articles/dataset/SData1/21295287) (BioProject accession no. PRJNA629478) (32) for BGC mining. The remaining three genomes are of archaeal origin and thus were not included. To compare the suitability of the 1,080 bacterial HQ MAGs for BGC mining based on estimates of complete BGCs, representative genomes of the phyla *Myxococcota* (583 genomes) and *Nitrospirota* (328 genomes) were selected from the GTDB-R202 data set (https://data.gtdb.ecogenomic.org/releases/release202/202.0/bac120_taxonomy_r202.tsv). Bacterial genomes from Liu et al. 2021 (26), Sharrar et al. (21), Crits-Christoph et al. (22), Chen et al. (24), and Bickhart et al. (28) were also included to investigate the effect of using long- or short-read sequence-based HQ MAGs on BGC mining and complete BGC recovery. For fair comparisons with MAGs generated from short-read data, HQ MAGs are defined using CheckM (66) with completion of >90% and contamination of <5%, and without the requirement of full-length rRNA genes (55). Using these criteria, we selected 73 HQ MAGs from Crits-Christoph et al. (22), 350 HQ MAGs from Sharrar et al. (21), 21 HQ MAGs from Chen et al. (24), 284 HQ MAGs from Bickhart et al. (28), and 153 HQ MAGs from Liu et al. (26). For consistency with the other studies, we reassessed the MAGs from Bickhart et al. (28) and Chen et al. (24) using CheckM *–*lineage_wf v1.1.3 to make the HQ MAG selection. When available, the assemblies in the fasta format (.fna) were downloaded from the NCBI GenBank database using ncbi-genome-download v0.3.1 (https://github.com/kblin/ncbi-genome-download). The MAGs and contig names from Chen et al. (24) and Sharrar et al. (21) were shortened to comply with the reannotation process.

### Taxonomic identification of HQ MAGs based on GTDB.

In order to characterize the distribution of various bacterial groups across the data sets, we assigned taxonomic definitions using the classify_wf workflow of the GTDB-tk v1.7.0 toolkit based on database version GTDB-R202 (see SData 1 at https://figshare.com/articles/dataset/SData1/21295287) (67, 68). These taxonomic classifications were used throughout the data analysis and data visualization. The phylogenomic tree generated as an output of GTDB-tk was processed further to select the subset of leaves with selected genomes used in the study (Fig. 4 and 5). GTDB-R207 was used to provide species-level assignments for Fig. S4.

### Genome mining analysis to detect BGCs in HQ MAGs.

All of the HQ MAGs in the data set were reannotated using Prokka v1.14.6, with Prodigal for open reading frame (ORF) detection and an E value of 1e^−5^ (69, 70). Secondary metabolite BGCs were detected across HQ MAGs using the genome mining software antiSMASH v6.0.1 (17). antiSMASH can detect up to 71 types of BGCs that are grouped into 8 major classes by BiG-SCAPE (18). The BiG-SCAPE classes “PKSI” and “PKSOther” were merged into “PKS,” and “Saccharide” was merged into “Other,” since the number of BGCs found in these classes was low (70 and 2, respectively). We analyzed and visualized the distribution of the number of BGCs across MAGs in R v4.1.2 using the package collection tidyverse v1.3.1 and the packages treeio v1.19.1 and ggtree v3.3.0 for phylogenetic tree manipulation and visualization of all figures showing phylogenetic trees (71, 72). Core genes of predicted-type terpene BGCs were annotated using DIAMOND v2.0.9 (73) for SData 3 (https://figshare.com/articles/dataset/SData3/21295320) against the NCBI nr database (downloaded 10 April 2021) using the command “diamond blastp –db nr.dmnd -q terpenes.fa -f 6 salltitles qseqid sseqid pident length mismatch qstart qend evalue bitscore -o out_terpenes_final.txt –max-target-seqs 1 -b12 -c1 –threads 80.” The functionally relevant genera from WWTPs were selected and their genus was assigned based on Singleton et al. (32) or manually assigned for the recently renamed *Tetrasphaera* species (54).

### Detection of GCFs in HQ MAGs.

To investigate whether the detected BGCs code for the biosynthesis of previously characterized secondary metabolites, we calculated a similarity network of all 4,238 HQ MAG BGCs and 1,926 known BGCs from the MIBiG data set (41). The similarity network was generated using BiG-SCAPE v1.1.2 with the hybrids_off option. Using the default cutoff value of 0.30 on the raw_distance metric, the BGCs were clustered into several gene cluster families (GCFs) and visualized using CytoScape (Fig. S3). Since there are no BGCs clustered together with the known BGCs from MIBiG, BiG-SLICE v1.1 was used to query all HQ MAG BGCs against the preprocessed result of ~1.2 million microbial BGCs as described in reference 50. We ran BiG-SLICE using the parameter *–*run_id 6, which queries against the BiG-SLICE run 6, with a clustering threshold of 900. This particular run is currently used by the BiG-FAM database v1.0.0 (https://bigfam.bioinformatics.nl/run/6). Only first hits are processed and visualized with CytoScape (Fig. S3 and S5).

### Code availability.

Detailed descriptions on how to acquire and preprocess these data sets are available as a Jupyter notebook and can be accessed from https://github.com/robertosanchezn/AS_hqMAGs/blob/main/jupyter_notebook/notebook2/01_other_MAG_dataset_table.ipynb. All steps in the annotation, genome mining, and GCF detection were managed using Snakemake v7.6.1 to ensure reproducibility (74). Other data and relevant code used for analyses can be found at https://github.com/robertosanchezn/AS_hqMAGs.

### Data availability.

Supplemental data files are available at https://doi.org/10.6084/m9.figshare.c.6237351.v1. The MAG data set from reference 30 can be accessed from the NCBI BioProject PRJNA629478. The MAG data set from Crits-Christoph et al. (22) is available from NCBI BioProject PRJNA449266. The MAGs from Sharrar et al. (21) are available at https://figshare.com/ndownloader/files/18105260. The subset of MAGs from Chen et al. (24) can be accessed from the MG-RAST database under project name mgp81948. The MAG data set from Bickhart et al. (28) can be accessed from https://zenodo.org/record/5138306. The MAG data set from Liu et al. (26) can be accessed from the NCBI BioProject PRJNA648801.

10.1128/msystems.00632-22.8 FIG S8 Number of BGCs of one study (*x* axis) found in a GCF of another study (*y* axis). Three different cutoff values were used for BiG-SCAPE clustering. Color indicates the percentage of the total number of BGCs shared between the studies. Download FIG S8, SVG file, 0.06 MB. Copyright © 2022 Sánchez-Navarro et al. 2022 Sánchez-Navarro et al. https://creativecommons.org/licenses/by/4.0/ This content is distributed under the terms of the Creative Commons Attribution 4.0 International license.
