# Supplementary figures and images for: Long-Read Metagenome-Assembled Genomes Improve Identification of Novel Complete Biosynthetic Gene Clusters in a Complex Microbial Activated Sludge Ecosystem
Source: mSystems. 2022 Nov 29;7(6):e00632-22. doi: 10.1128/msystems.00632-22 (PMC9765116; doi:10.1128/msystems.00632-22)

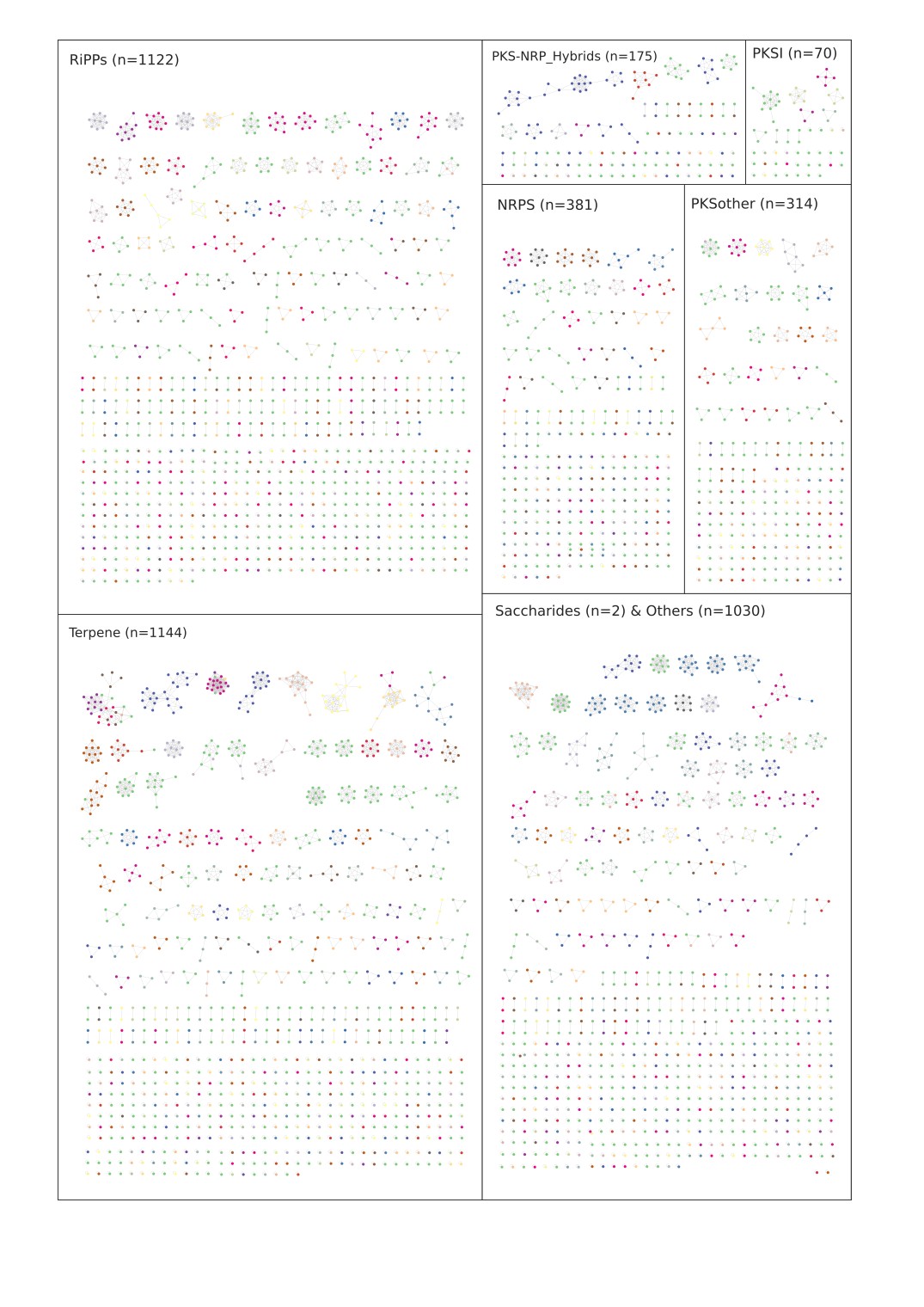

Supplement: FIG S3 [file msystems.00632-22-s0003.jpg]
